# Supplementary figures and images for: Disease Resistance and Molecular Variations in Irradiation Induced Mutants of Two Pea Cultivars
Source: Int J Mol Sci. 2022 Aug 8;23(15):8793. doi: 10.3390/ijms23158793 (PMC9369183; doi:10.3390/ijms23158793)

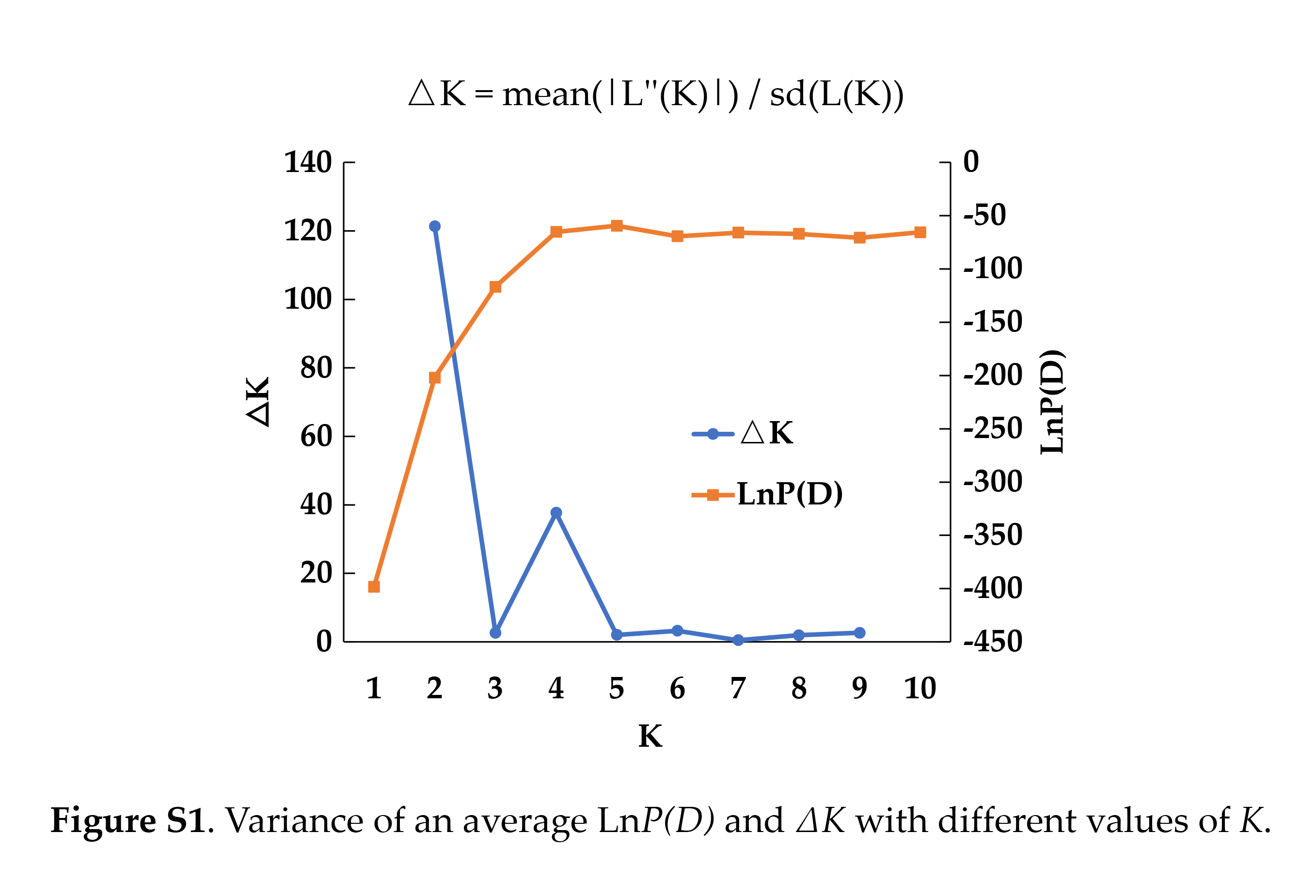

Supplement: Supplementary file 1 [file ijms-23-08793-s001.zip › Supplementary Figure S1.png]
